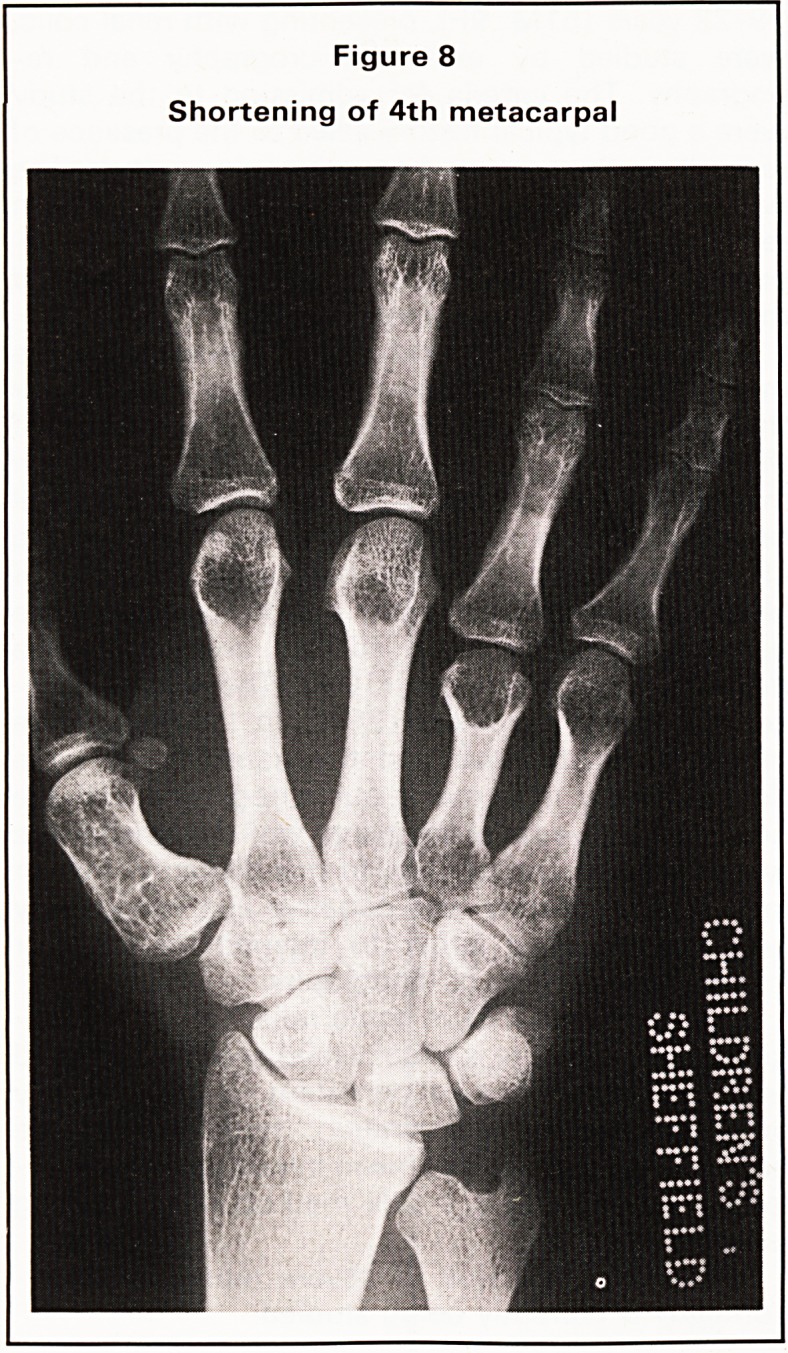# South West Radiologists Association

**Published:** 1983-10

**Authors:** 


### South West Radiologists Association

### Annual Meeting at Bristol Royal Infirmary 12th March 1983

**Ian Gordon Memorial Lecture**

**'THE LONG AND THE SHORT OF IT'— RADIOGRAPHIC ASPECTS OF BONE GROWTH**
**The First Ian Gordon Memorial Lecture given by Dr. Keith Levick of Sheffield**

Ian Ronald Simpson Gordon was appointed Consultant Radiologist to the United Bristol Hospitals in 1954. He had previously worked as a physician and gained his Membership of the Royal College of Physicians and a Doctorate in Medicine from his University of Cambridge. After coming to Bristol to train in radiodiagnosis, his particular interest in children's problems led him to take charge of the X-ray Departments at the Bristol Children's Hospital and Bristol Maternity Hospital.

Ian Gordon rapidly established an international reputation as a paediatric radiologist and he was a founder-member of the European Society for Paediatric Radiology in 1964. Together with Dr. Frank Ross, he wrote an eminently readable book on diagnostic radiology in paediatrics which is the standard work on this subject in the United Kingdom.

Two recollections of Ian Gordon illustrate some of his important qualities. First, his determination. With two young children to transport along his favourite precipitous footpaths, he and his wife tied a rope to the front of the pram and then traversed the same routes as they had done before the family arrived! Second, the confidence and devotion he inspired in all who worked with him. When a nervous child or an infant with difficult veins was due for examination, they were added to Ian's already long list as 'specially selected for Dr. Gordon'. This, then, is the man whom we honour today in this First Memorial Lecture.

I have chosen, as my subject, to examine some of the many aspects of bone growth which we may follow on radiographic examination.

*Foetal detection*

At about the fifth week of foetal life, bone begins to be formed by a condensation of primitive mesenchyme. By about the twelfth week of foetal life it is possible, under ideal circumstances, to see parts of the foetal skeleton on an oblique view of the maternal pelvis. At this stage of pregnancy we may see the beginnings of the vertebral column, early rib development, long bones and the base of the skull. During the succeeding weeks of pregnancy, bone growth takes place in two ways, by enchondral ossification and by membranous bone formation, so that at term we can identify all major parts of the skeleton, and comment on foetal maturity according to the development of various epiphyses. However, Rohan Williams, writing in the 1957 edition of 'Radiology by British Authors', was already drawing attention to the possible hazards of radiation to the foetus after a preliminary communication from Dr. Alice Stewart to the Lancet in 1956. Subsequently, the indications for radiography during pregnancy became more stringent and much of the expertise built up over the years in studying foetal growth in this way has become of historic interest. However, I should like to explore some of the residual indications for foetal radiography at the present time.

*Foetal abnormality*

A combination of ultrasound skills and chemical pathology now provide much information about possible abnormality. An early example from our ultrasound examinations in Sheffield shows an 18 week pregnancy with an abnormally small foetal skull associated with a fluid-containing area. Whilst we were debating the significance of this back in 1966, a spontaneous abortion provided the answer which is, of course, an encephalocele associated with a small skull. Ultrasound examination has proved of increasing value in confirming spinal dysraphism, perhaps suspected by an abnormal alpha foeto-protein level, in looking at the foetal head and at development of the gastro-intestinal and urinary tracts. Until recently there has been less confidence in studying limb growth in the foetus. It is here that foetal radiography has remained a useful technique. I should like to include two examples of this. The first concerns a family whose first child, a boy, was born with no thumb development and evidence of a cardiac septal defect. On enquiry, his father was found to have abnormally short thumbs, but no other problem. A diagnosis of Holt Oram syndrome was made, and the child treated by a pollicisation procedure. The parents enquired as to the risks of deformity in any subsequent children. They were told that this condition has a variable penetrance and that all degrees of problem could appear, the most extreme being a failure of upper limb development. It was decided that any future pregnancy should be monitored with a view to possible termination if serious foetal malformation was found. Under these circumstances we find that a single radiograph at 20 weeks will usually provide the relevant information. In the examination of the second pregnancy, there was no evidence of upper limb development apart from a small bone near the shoulder (Figure 1). The pregnancy was terminated. My second example concerns a second pregnancy where the mother had previously delivered a still-born deformed foetus at about 32 weeks. From the description, we suspected that this had been a thanatophoric dwarfism problem and it was decided to monitor progress in the second pregnancy. The foetal radiograph showed characteristic features of this form of dwarfism with short limb bones, curvature of the femora, flat vertebral bodies and shortened ribs. Post-termination radiograph confirms a diagnosis of thanatophoric dwarfism (Figure 2).

**Figure 1**

**Oblique abdomen in late pregnancy. There is a single foetus and only one rudimentary upper limb bone can be identified**

The advent of real ultrasound techniques means that limb development can now be studied more adequately and Stuart Campbell has provided tables of normal femoral length at various ages. So we may attempt to identify short limb dwarfism by ultrasound methods, perhaps using a radiograph as final confirmation only.

**Figure 2**

**Post-partum radiograph of same infant as Figure 1 showing thanatophoric dwarfism**

*Disorders of bone growth*
To return to a more general lack of bone growth and its disorders in the orderly process of enchondral ossification at the growing end of a long bone, cartilage cells form orderly columns, become degenerate and calcified, and are then removed and replaced by osteoid and mineralised bone. Defects of growth at various levels and stages produce a variety of well-known bone dysplasias. For example, slow production of cartilage cells of poor quality is associated with achondroplasia; this type of hypoplasia contrasts with an abnormal proliferation of cartilage cells producing an enchondroma.

*Chondrodystrophy*
The study of dwarfism in general has a fascination for us, and even if we confine ourselves to the field of short limb dwarfism in the neonate, we need to consider a dozen or more possibilities in the differential diagnosis. This young man demonstrates the clinical features of a chondrodystrophy, and the radiograph of his pelvis and femora shows the clinical features of achondroplasia (Figure 3). The pelvis is the key to a several chondrodystrophies with its flat acetabular roofs, bone spurs at the inner and

**Figure 3**

**Achondroplasia. A.P. radiograph of pelvis and femora**

**Figure 4**

**Achondroplasia. Secondary hydrocephalus due to under-developed cartilage growth around the foramen magnum**

outer margins and narrow sarco-sciatic notches. The problems of cartilage growth may also become severe in the skull base where under-development can lead to construction around the foramen magnum with secondary hydrocephalus and, in some instances, damage to the upper end of the spinal cord (Figure 4). Another example of the chondrodystrophy pelvis is found in metatrophic dwarfism. The same features of the flattened acetabular roof, bone spurs and the small sacro-sciatic notches are again found, femoral deformity is likened to a halberd. In this dystrophy, we also find a narrow thoracic cage and marked flattening of the vertebral bodies as in thanatophoric dwarfism.

At this stage, we may be tempted to ask, 'Does this offer us more than a gamesmanship situation, where our reports contain a classically derived diagnosis but little else of value?' The answer is there are two important sequelae of recognising a particular form of dwarfism in a newborn baby. First, we may predict physical and mental development of the infant. For example, in metatrophic dwarfism, mental development is usually normal, but there is increasing physical deformity due to a developing kypho-scoliosis in addition to the already short limbs. Secondly, we may be able to advise the parents about the risk of further affected children. For example, metatrophic dwarfism has an autosomal recessive inheritance and there is a one-in-four risk of further affected pregnancies. Thanatophoric dwarfism also is autosomal and recessive, although there is some argument as to whether this is only true if a clover leaf skull deformity is present.

a. *Osteogenesis Imperfecta*

I would now like to turn to one of the other possible causes of short limb dwarfism in the newborn. When we review a radiograph which shows gross deformities in the long bones, ribs and skull, many of which appear to be due to healing fractures, we can all identift the severe neonatal form of osteogenesis imperfecta without difficulty. This condition is, of course, based on collagen formation, so that the bone lacks a normal scaffold for its formation. The survivors of this group suffer from severe skeletal deformity, and may require medullary pinning of most of the long bones. The tarda variety of this condition is well known, with its rather unreliable finding of blue sclera due to a thin layer of scleral collagen allowing underlying pigment to show through. There is a 'no-man's land' which lies between these two well-recognised forms, so that in proceedings against parents suspected of baby battering, a defence of 'fragile bones' may be entered. In some instances, this unhappy group of infants will show a characteristic mixture of soft tissue injuries, fractures of varying ages, particularly in the ribs and metaphyses of long bones, sometimes associated with serious skull injuries and underlying subdural haematomata. In these circumstances, we would have little difficulty in identifying a battered baby (Figure 5).

**Figure 5**

**Non-accidental injury. Serial chest radiographs showing multiple fractures**

b. *Non-accidental Injury*

Let us consider a much more difficult problem, however: an infant brought in with a fractured clavicle, scattered bruising of various ages, but no evidence of other bone trauma, either old or recent, on a skeletal survey. A family history of fragile bones is claimed by the parents. We may examine the long bones carefully for evidence of low bone density, over-constriction of the diaphyses and sometimes minimal metaphyseal flaring. We may look at the skull for evidence of multiple Wormian bones. All too often these signs are equivocal. Attempts to examine the quality of bone collagen are not only known to be difficult to interpret, but also involve a further assault on the child in the need to obtain a tissue sample. Measurements of hydroxyproline excretion in the urine also seem to be unreliable as an index of abnormal collagen metabolism.

The radiographic findings are, of course, only part of a much larger situation where the social history and clinical examination are important. The radiologist must look carefully for signs of battering, but beware of over-interpreting minimal signs of skeletal development.

*Assessment of bone age*

Incompatibilities between chronological age and skeletal size often lead to a request for an estimation of bone age. Bone age, conventionally estimated from a radiograph of the wrist and hand, may be measured in two ways. The Tanner Whitehouse System II is recognised as being the most accurate available. This entails the comparison of twenty separate bones in the wrist and hand, with the various developmental stages of each of these bones, and produces an averaged result at the end of the survey. Most radiologists would take 20 to 30 minutes to complete this accurately, and I suspect that for this reason the Greulich & Pyle Atlas is more popular in most X-ray Departments. A picture-matching process with Greulich & Pyle is usually complete in about 5 minutes. We must accept that in situations where the effects of growth hormone or thyroxin therapy are being monitored, there is a need for the Tanner Whitehouse II, but we also need to know how accurate a single assessment by Greulich & Pyle's method would be when we assess the bone age of a child of short stature from a radiograph coming through in our general reporting.

Last year, we carried out a comparison between the two methods as used in the Sheffield Children's Hospital, Greulich & Pyle being the method used in the X-ray Department and the Tanner Whitehouse System being used in the Auxology Unit of the University Department of Paediatrics. The results may interest you,either as a possible defence of Greulich & Pyle or as an indication of the likely degree of inaccuracy this method may bring about. In our survey, we took the wrist and hand radiographs of 100 children who had been referred sequentially for Tanner Whitehouse II estimations of bone age in the University department. In the first instance, all these films would have been seen by a radiologist and Greulich & Pyle estimate of bone age issued. These films were first reviewed by two independent observers and a new bone age estimate was made without reference to previous reports or clinical indications. I am happy to say that both observers showed a fairly close degree of correlation, here are their results clustered around the line of identity. We also carried out a type of quality control, by matching the mean of the two observers' reports against the original report, which may have been given by one of two consultants or a rotating senior registrar. We may now compare the mean results from the two observers with the reports of the same children from the Tanner Whitehouse system (Figure 6). Very few of the results lie above the line of identity. Further analysis of the results shows that the Greulich & Pyle estimates are approximately 0.8 of a year, i.e. about $8\frac{1}{2}$ months younger than the Tanner Whitehouse bone ages. This probably reflects the selected pre-war American population of children referred to a paediatrician, who form the basis of Greulich & Pyle's studies and the greater socio-economic scatter of the children used to develop the Tanner Whitehouse systems.

Our conclusions may be summarised as follows: first, that we recognise a quantitative but not qualitative difference between the two systems; second, that the Tanner Whitehouse system gives a more advanced bone age and, lastly, that the Greulich & Pyle method is adequate for initial diagnosis, but not adequate for the close follow-up of children receiving growth hormone and other endocrine therapy.

*Trauma and bone growth*

The effects of trauma on bone growth are widely recognised, as in the advantage that continued growth and remodelling gives to a child in recovering

**Figure 6**

**Assessment of bone age. Comparison of Greulich & Pyle method with Tanner Whitehouse method. See text for explanation**

**Figure 7**

**Irregular carpal bone ossification following previous crush injury**

from deformity which may follow some fractures. Angulation of a long bone in the plane of the major movement of a joint is known to be subject to a slow proces of correction, as we may see in this 12-month follow-up of a healed lower radial fracture with angulation. Bone growth may also be affected by iatrogenic trauma, as we see in a short humerus resulting from previous radiotherapy. One poorly understood field, however, remains that of damage to cartilagenous centres before ossification has commenced. Here we see the wrist radiograph of a child with a history of a crush injury some years previously (Figure 7). We may just notice the present reason for the radiograph on the edge of the film, but we were intrigued by the irregular appearance of carpal bone ossification, which did not seem to relate to any recent injury, to any generalised abnormality of ossification, or indeed to any restriction of movement or function of the wrist and hand. We were forced to conclude that this must be a sequel of the injury recorded some years previously. But how rarely do we see evidence of abnormal growth in a bone where the history suggests that it could have been damaged at the cartilagenous centre, and we must believe that most of the centres at this stage are able to reform and grow normally, despite injury at this stage.

I should like to end this look at bone growth in a Paediatirc X-ray Department by identifying a few of the many conundrums that remain. A baby, with the classical clinical appearances of hypothyroidism or cretinism, shows a characteristic lesion at the dorsolumbar junction. The body of the first lumbar vertebra shows anterior dysplasia and a beak may arise at the lower border, or may also be more or less central. We know that this type of spinal deformity can also occur in the Hunter Hurler type of mucopolysaccaride disease. Why is the dorsolumbar junction particulary liable to abnormality in these two diverse conditions?

Metacarpal shortening may be single or multiple (Figure 8). It is well known that a short fourth metacarpal may be a normal finding in a small percentage of the population but its association with Turner's syndrome is well known also. Shortening of the one-four-five variety has been associated with the unusual and interesting group of conditions called the pseudo-hypoparathyroidisms, often quoted as a good example of a failure of end organ response to a normal production of parathormone. Up to three or four years ago, I would have confidently made a diagnosis of this condition on seeing such an appearance. Now, however, there are a growing number of brachydactyly syndrome, associated with changes in the renal and cardiovascular systems, though I am sure more are yet to be discovered. Perhaps we should return to our earlier premise, that the reason for identifying such unusual syndromes must be constructive and give a forecast of the child's likely progress and the genetic hazards of further involved children.

**Figure 8**

**Shortening of 4th metacarpal**

In conclusion, may I say how much pleasure I have in paying tribute to Ian Gordon. I should like to thank Dr. Frank Ross and Mrs. Kilby for the help they have given me and to thank the South West Radiologists Association most sincerely for the honour they have done me in inviting me to be their speaker.

### Abstracts of Papers

### RESULTS AND COMPLICATIONS OF PERCUTANEOUS TRANSLUMINAL ANGIOPLASTY

**W. D. Jeans, Bristol**

The technique of dilatation of narrowed and occluded segments of artery by an intra-arterial catheter having a distensible balloon in its distal end was first described in 1974 by A. Gruntzig. Its use has gradually increased and is now widely accepted. One report (Motorjeme et al., 1980) suggests that some 70% of patients with symptoms of arterial insufficiency may be suitable for dilatation either alone or in conjunction with surgery. The results in the first 52 patients in whom the technique was attempted in Bristol confirmed the reported indications and complications. 52 patients were catheterised and in 12 the lesion could not be passed or was not dilated. 70% of those dilated had a successful and persisting response to the technique. Success is more likely if there is no calcification present; if there is a stenosis or occlusion less than 12 cm. in length and if the lesion is in the iliac or superficial femoral artery. Complications include haematoma in the groin (worse because patients are heparinised), embolism and dissection of the vessel being dilated. These complications make it essential for the procedure to be done in association with vascular surgeons.

### RADIONUCLIDE EVALUATION OF ACUTE SCROTAL DISEASE

**R. A. Nakielny, Bristol**

$^{99}Tc^{m}$ Sodium Pertechnetate scintigraphy is an established method for evaluating organ perfusion and can be applied to the investigation of suspected testicular ischaemia due to acute torsion. One hundred and six consecutive scrotal investigations have been analysed qualitatively and quantitatively using a computer based comparison of the perfusion slopes over each testis. Decreased vascularity on the symptomatic side only occurs in torsion and quantitative analysis is needed to detect it reliably. Increased vascularity on the symptomatic side can usually be detected qualitatively and occurs in orchitis, tumours, trauma and resolved torsion. Distinction between the cause of increased vascularity is aided by the clinical data.

A halo of increased activity surrounding a relatively cold centre occurred in chronic torsion, some tumours and hydroceles. The recognition of chronic torsion is vital, and can be achieved qualitatively and quantitatively.

The test is both sensitive and specific for acute torsion but its usefulness is limited if it is not available at all hours. The test is useful also in confirming a diagnosis that does not require emergency operation, and in identifying chronic torsion which is an indication for contralateral orchidopexy.

### IMPROVEMENTS IN THE ANGIOGRAPHIC DEMONSTRATION OF CAROTID BIFURCATION LESIONS

**Stephenie Mackenzie, W. D. Jeans and R. N. Baird, Bristol**

Experimental studies of casts of postmortem carotid bifurcations and of cineangiograms of the carotid formed the basis for a prospective study in which three views were taken of each carotid bifurcation in a series of 101 patients being investigated by catheter for transient ischaemic attacks. Analysis of the results showed that 12 internal carotid arteries were occluded, one patient having a bilateral occlusion, and 53 bifurcations were normal although only 13 patients had normal bifuractions on both sides. Complicated atheroma was shown significantly more often ($p < 0.01$) on two or three views when compared to simple atheroma, suggesting that this technique of three views showed small lesions. The view which would show the disease could not be predicted, suggesting that the three views were essential to be sure that an accurate statement of the presence or absence of disease could be made.

### THE VALUE OF RENOGRAPHY IN ACUTE RENAL COLIC

**C. Davies, Bristol**

Preliminary results were presented on one aspect of a long-term study of acute renal colic being conducted by the Departments of Urology and Radiodiagnosis.

In Bristol the conventional management of urinary tract stones is conservative. Among the questions raised in this context are the effects of such management on renal function and the need for emergency urography. In the present study, 60 patients aged 19–22 years (51M : 9F), presenting with renal colic, were studied by excretion urography and renography. The criteria for admission to the study were a good typical history allied to the presence of red cells in the urine and negative urine culture. The well-recognised urographic signs of abnormality in renal colic were sought by a radiologist independently of analysis of serial renography images and measurement of mean transit times.

In the symptomatic kidneys the average of the mean transit times (6.3 min) was substantially higher than that in normal kidneys (2.2 min). The severity of the abnormality varied considerably, being over ten minutes in six instances (10%) and within the normal range in 19 patients (32%). In many of the latter the clinical abnormalities were abating at the time of renography. Both urography and renography were positive in 37 instances (6.1%); and both were negative in six instances (10%). There was a discordance in 17 instances (29%). In 13 of these the urogram was positive and the transit times were normal. Delay in carrying out renography and the need to observe serial images as well as transit times were the main contributors to this disparity. In four instances urography was negative and renography was positive, due either to intermittent colic or increased sensitivity of renography.

It is concluded that in acute renal colic the renographic abnormality is usually mild and transient and sometimes severe but not persistent: renography may be influenced by dehydration: serial images are necessary to supplement mean transit time measurements: renography is a safe method of monitoring renal function.

The predictive value of severe renographic abnormality is currently being studied.

## Figures and Tables

**Figure 1 f1:**
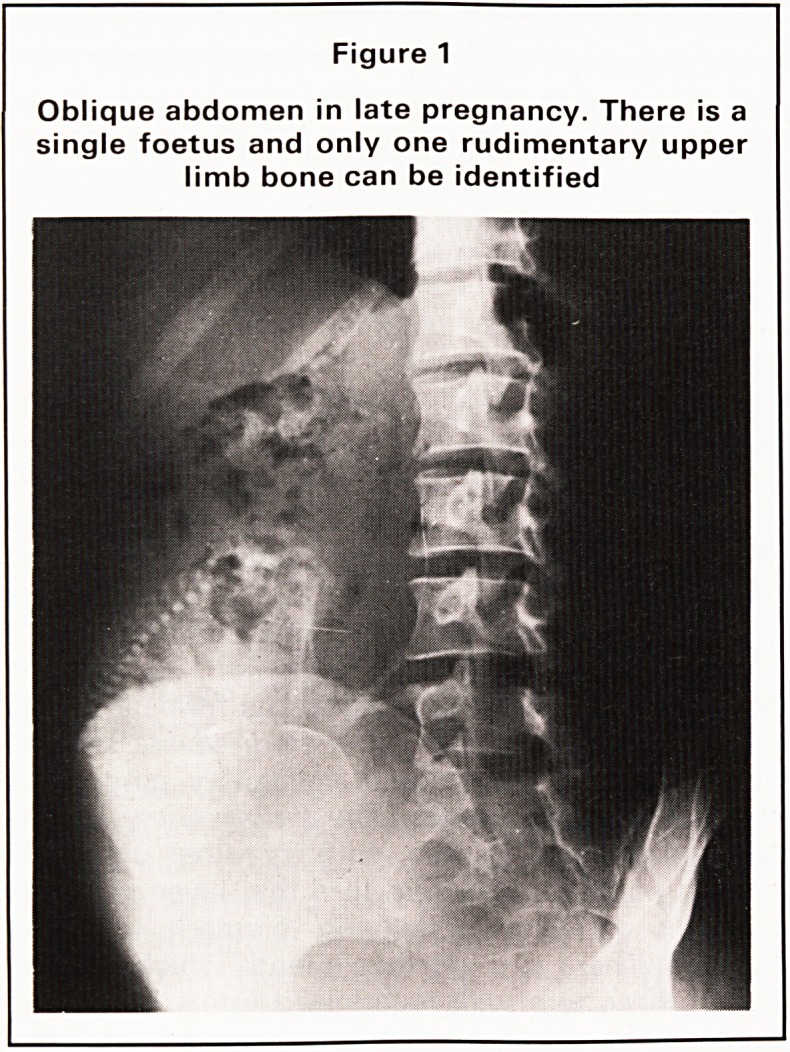


**Figure 2 f2:**
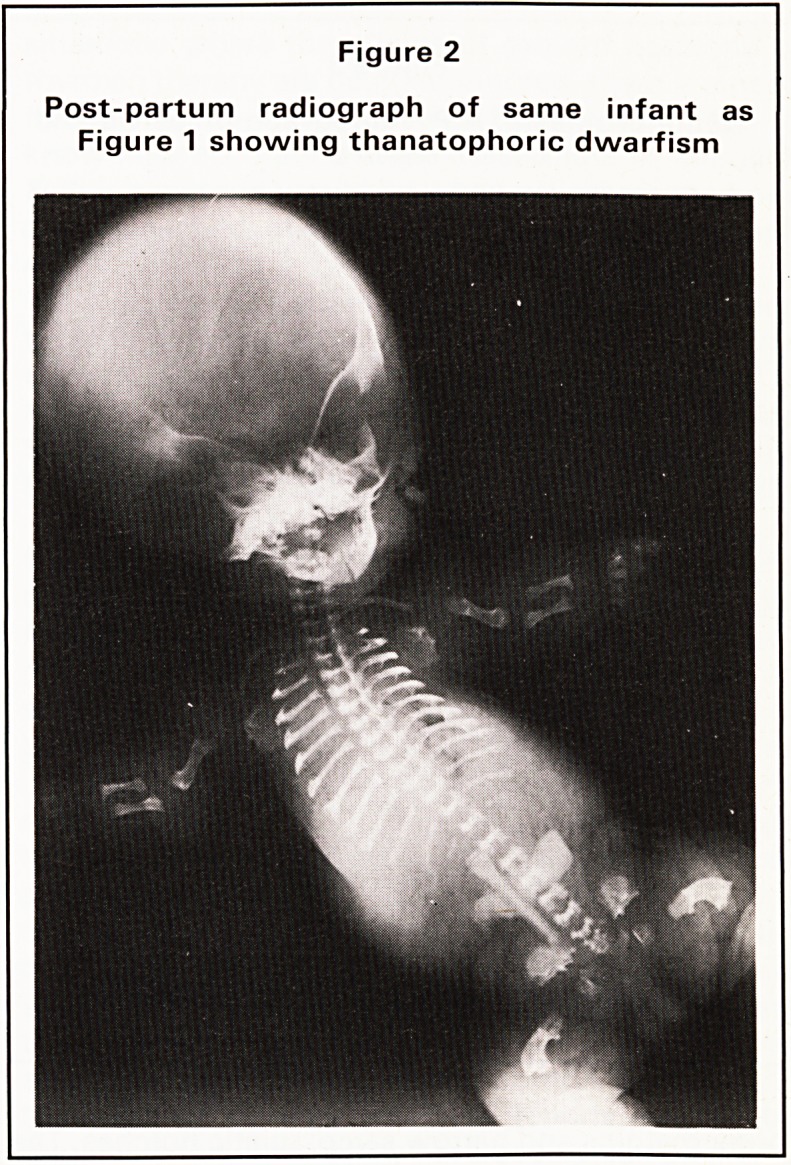


**Figure 3 f3:**
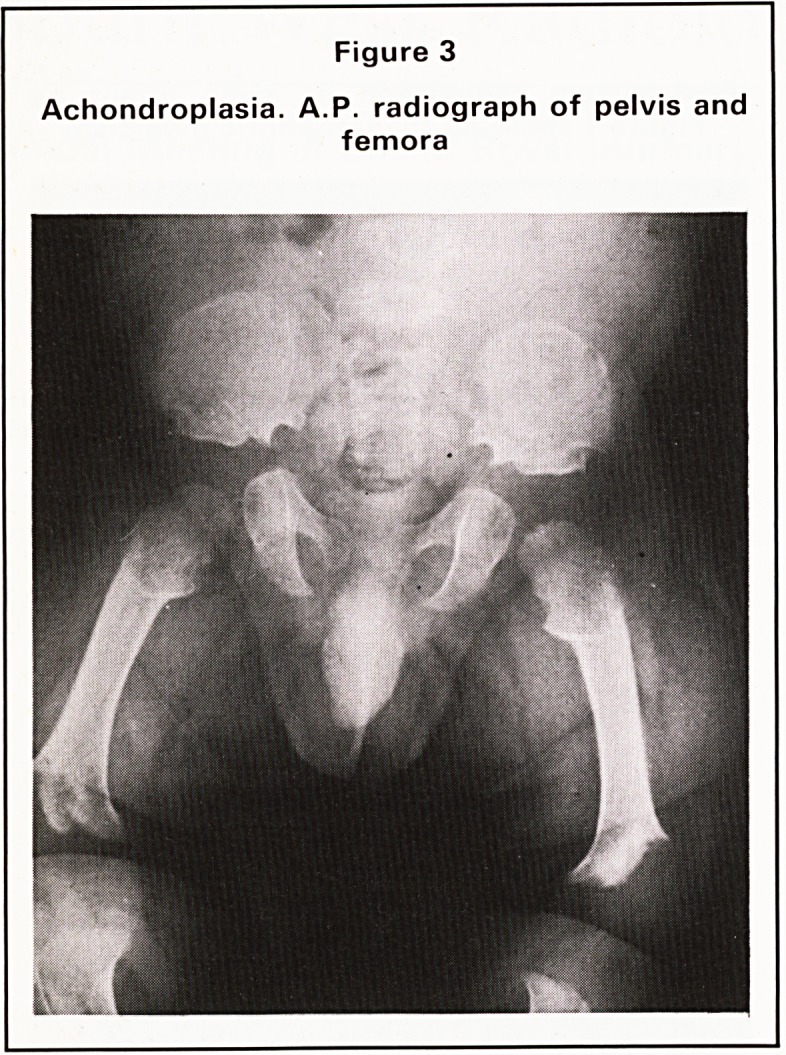


**Figure 4 f4:**
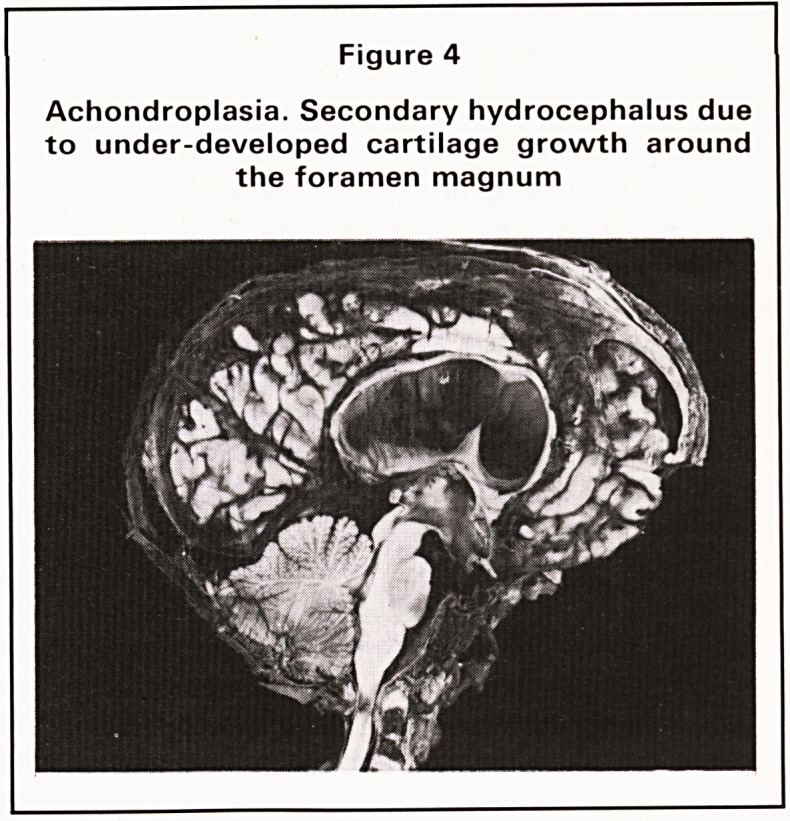


**Figure 5 f5:**
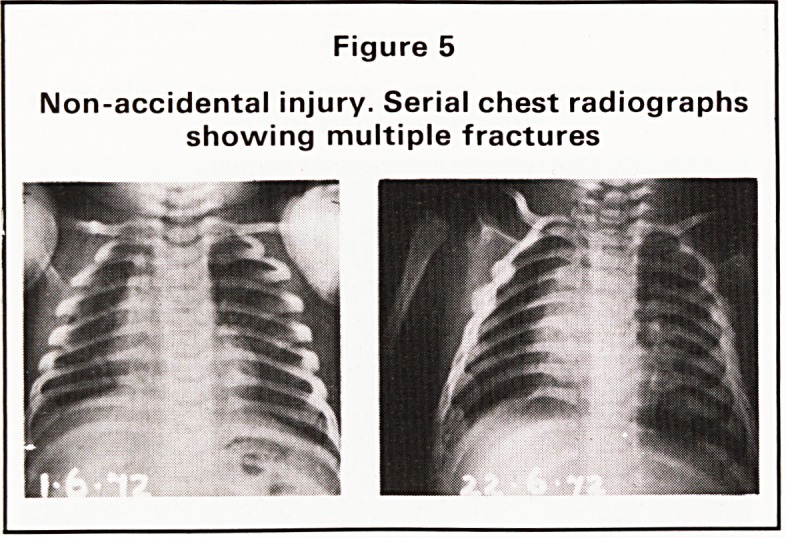


**Figure 6 f6:**
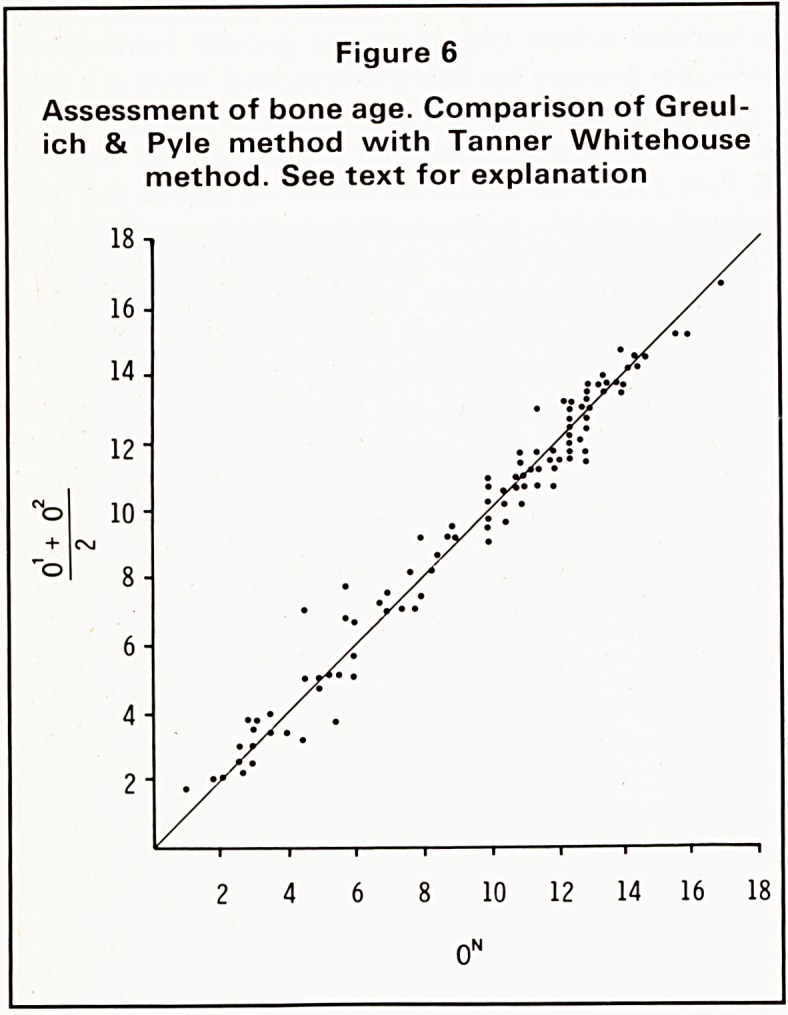


**Figure 7 f7:**
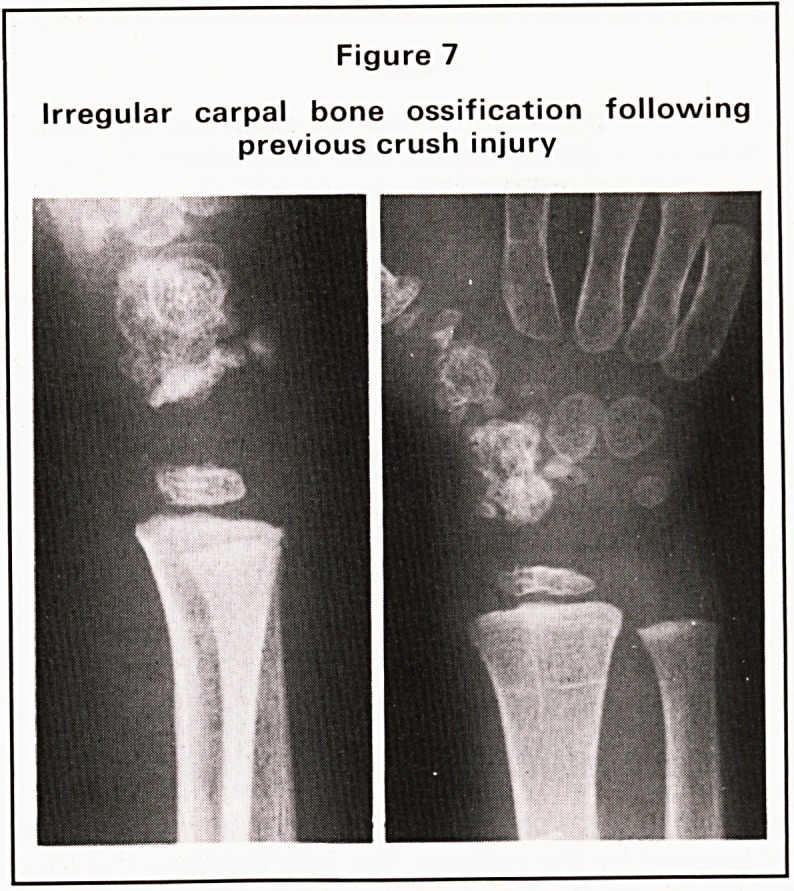


**Figure 8 f8:**